# Characterization of SARS-CoV-2 Omicron BA.2.75 clinical isolates

**DOI:** 10.1038/s41467-023-37059-x

**Published:** 2023-03-23

**Authors:** Ryuta Uraki, Shun Iida, Peter J. Halfmann, Seiya Yamayoshi, Yuichiro Hirata, Kiyoko Iwatsuki-Horimoto, Maki Kiso, Mutsumi Ito, Yuri Furusawa, Hiroshi Ueki, Yuko Sakai-Tagawa, Makoto Kuroda, Tadashi Maemura, Taksoo Kim, Sohtaro Mine, Noriko Iwamoto, Rong Li, Yanan Liu, Deanna Larson, Shuetsu Fukushi, Shinji Watanabe, Ken Maeda, Zhongde Wang, Norio Ohmagari, James Theiler, Will Fischer, Bette Korber, Masaki Imai, Tadaki Suzuki, Yoshihiro Kawaoka

**Affiliations:** 1 Division of Virology, Institute of Medical Science, University of Tokyo, Tokyo, 108-8639 Japan; 2 The Research Center for Global Viral Diseases, National Center for Global Health and Medicine Research Institute, Tokyo, 162-8655 Japan; 3 Department of Pathology, National Institute of Infectious Diseases, Tokyo, 162-8640 Japan; 4 Influenza Research Institute, Department of Pathobiological Sciences, School of Veterinary Medicine, University of Wisconsin-Madison, Madison, WI 53711 USA; 5 Disease Control and Prevention Center, National Center for Global Health and Medicine Hospital, Tokyo, 162-8655 Japan; 6 Department of Animal, Dairy, and Veterinary Sciences, College of Agriculture and Applied Sciences, Utah State University, Logan, UT 84322 USA; 7 Department of Virology 1, National Institute of Infectious Diseases, Musashimurayama, Tokyo 208-0011 Japan; 8 Center for Influenza and Respiratory Virus Research, National Institute of Infectious Diseases, Musashimurayama, Tokyo 208-0011 Japan; 9 Department of Veterinary Science, National Institute of Infectious Diseases, Tokyo, 162-8640 Japan; 10 Space Data Science and Systems, Los Alamos National Laboratory, Los Alamos, NM 87545 USA; 11 Theoretical Biology and Biophysics, Los Alamos National Laboratory, Los Alamos, NM 87545 USA; 12 New Mexico Consortium, Los Alamos, NM 87545 USA; 13 The University of Tokyo, Pandemic Preparedness, Infection and Advanced Research Center, Tokyo, 162-8655 Japan

**Keywords:** SARS-CoV-2, Viral pathogenesis

## Abstract

The prevalence of the Omicron subvariant BA.2.75 rapidly increased in India and Nepal during the summer of 2022, and spread globally. However, the virological features of BA.2.75 are largely unknown. Here, we evaluated the replicative ability and pathogenicity of BA.2.75 clinical isolates in Syrian hamsters. Although we found no substantial differences in weight change among hamsters infected with BA.2, BA.5, or BA.2.75, the replicative ability of BA.2.75 in the lungs is higher than that of BA.2 and BA.5. Of note, BA.2.75 causes focal viral pneumonia in hamsters, characterized by patchy inflammation interspersed in alveolar regions, which is not observed in BA.5-infected hamsters. Moreover, in competition assays, BA.2.75 replicates better than BA.5 in the lungs of hamsters. These results suggest that BA.2.75 can cause more severe respiratory disease than BA.5 and BA.2 in a hamster model and should be closely monitored.

## Abstract (web summary)

Omicron subvariants may differ in their replicative fitness and their potential to cause more severe disease. In this study, the authors characterized Omicron BA.2.75 in a hamster model and found that it replicates more efficiently in the lungs than BA.2 and BA.5.

## Introduction

**Fig. 1:**
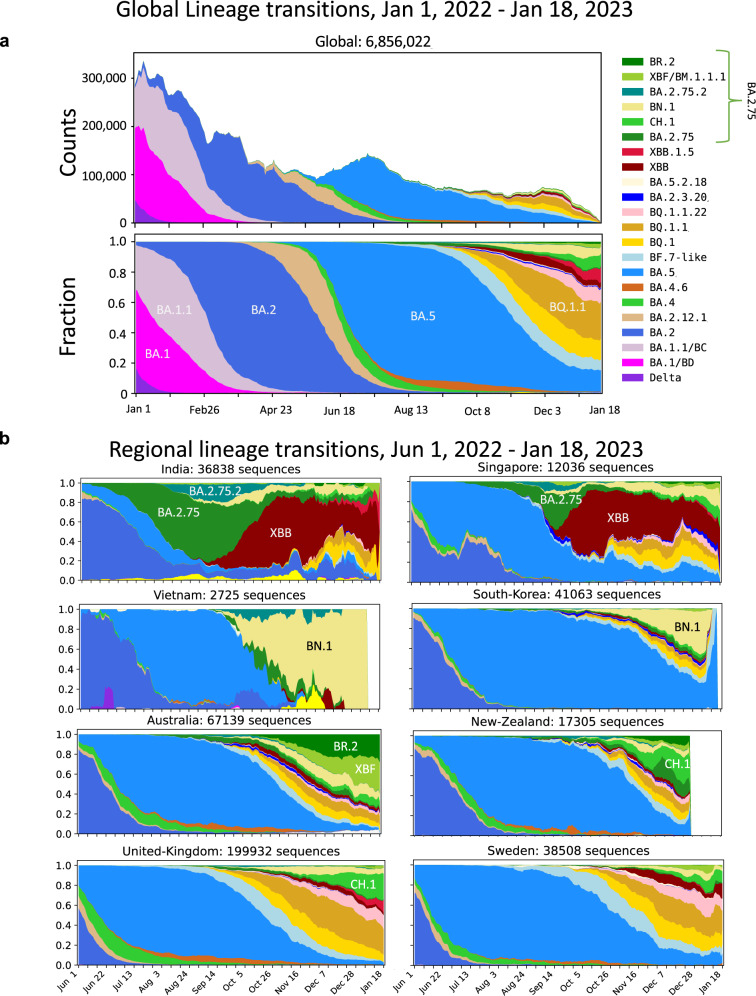
Pango lineage dynamics. **a** Global variant dynamics between Jan. 1, 2022 and Jan. 18, 2023. Omicron variants have been through waves of global dominance since Omicron began to spread in late 2021. BA.1 and BA.1.1 very rapidly replaced Delta globally. BA.2 then replaced BA.1 as the globally dominant form. Within the BA.2 lineage, BA.2.12.1 began to expand in North America, but BA.2, including the BA.2.12.1 sublineage, was soon replaced by BA.5 in a nearly global selective sweep. Currently, BA.5 is being replaced by a very complex array of distinct lineages that have evolved from either BA.2, BA.5, BA.2.75, or expanding recombinant lineages. (Pango lineage designations indicate recombinant lineages by starting their designation with an X.) Distinct common contemporary BA.2.75 sublineages (with associated forms of Spike defined in Supplementary Fig. 1) are considered individually. All BA.2.75-related sublineages are labeled in shades of green and arranged at the top of the graphs. **b** Examples of countries illustrating the increase in prevalence of BA.2.75-related lineages. India and Singapore are shown because they were among the first countries to transition to a highly prevalence of BA.2.75 in the summer of 2022; in both cases, the XBB recombinant lineage soon transitioned to the most prevalent form, by October of 2022, but currently BA.2.75 sublineages with additional mutations are once again beginning to expand in these countries. Vietnam and South Korea are displayed because they have a high prevalence of the BA.2.75 sublineage BN.1. Australia and New Zealand each have an array of BA.2.75 lineages that in combination dominate recent sampling. The UK and Sweden are both examples of countries were BA.2.75 sublineages are increasingly sampled. Supplementary Figs. 2 and 3 show that the two most common sublineages of BA.2.75, CH.1 and BN.1, are increasingly sampled in most countries once they are established, and illustrate the analysis used to identify the countries of interest for display here. All data are from GISAID; the illustrations were created by using the “Embers” web-based tool at cov.lanl.gov^26^.

Severe acute respiratory syndrome coronavirus 2 (SARS-CoV-2), first detected in China at the end of 2019, is responsible for COVID-19, which is associated with mild to severe symptoms ranging from cough and fever to severe pneumonia and death. Over two years have passed since the World Health Organization (WHO) declared COVID-19 a pandemic (https://covid19.who.int/). Yet, SARS-CoV-2 still imposes huge public health and economic burdens worldwide. The Omicron variant (Pango lineage B.1.1.529) emerged at the end of 2021 and has since evolved into complex sublineages. Three major Omicron lineages have serially transitioned as globally dominant forms: first BA.1, then BA.2, and then BA.5 (Fig. 1a). BA.5 began to expand in India in May 2022, when BA.2.75 (a sublineage of BA.2) was first detected there (Fig. 1b); the BA.2.75 subvariant rapidly became the dominant form in India and increased in prevalence in other regions suggesting that it had a selective advantage relative to BA.5. The WHO has categorized BA.2.75 as an Omicron subvariant under monitoring.

BA.2.75 has continued to diversify, and as of this writing (2022/01/28) BA.2.75 has been further subdivided into 111 Pango sublineages (https://github.com/cov-lineages/pango-designation/blob/master/lineage_notes.txt#L807). Here, we characterize the virological behavior of the ancestral form of the BA.2.75 lineage as it first emerged in India; although this ancestral form is no longer circulating, it forms the basic backbone of all later Pango BA.2.75 sublineages (Supplementary Fig. 1a). The newly emergent forms within the BA.2.75 lineage remain globally relevant in January 2023, and global sampling reflects a remarkable diversity of forms descended from BA.2, BA.5, and BA.2.75 co-circulating with distinct regional patterns (Fig. 1, Supplementary Fig. 1a).

Compared with the ancestral strain Wuhan/Hu-1/2019 (Pango lineage A; Wuhan Hu-1), the Omicron BA.1 virus had more than 30 amino acid differences in the spike protein of SARS-CoV-2 including insertions and deletions (Supplementary Fig. 1a) [by comparison, Delta (Pango lineage B.1.617.2) differed from the ancestral Wuhan Hu-1 by only 11 amino acids in its Spike]^1^. BA.2 differed from BA.1 at 27 Spike positions, and BA.5 differs from BA.2 by 5 amino acids in the S protein (Supplementary Fig. 1a). We demonstrated that the pathogenicity of BA.1 and BA.2 sublineage viruses is comparable in animal models and attenuated compared with previously circulating variants of concern (VOCs), consistent with clinical data in humans^2,3^. In addition, our recent data suggest that BA.4 and BA.5 have similar pathogenicity to that of BA.2 in rodent models^4^. SARS-CoV-2 initiates infection through the binding of the receptor-binding domain (RBD) of its spike protein to host cell surface receptors [i.e., human angiotensin-converting enzyme 2 (hACE2)]. BA.2.75 differs from that of BA.2 by nine amino acids in the Spike, including four in the RBD (i.e., G339H, G446S, N460K, and the wild-type amino acid at position Q493). Recent studies reported that the RBD of BA.2.75 has a higher binding affinity for hACE2 than that of BA.2^5,6^, raising the possibility that this property may increase the replicative ability and/or pathogenicity of BA.2.75. Moreover, in addition to the substitutions in the RBD, there are several amino acid differences in the other viral proteins of BA.2.75, which may also alter its replicative capability and pathogenicity (Supplementary Fig. 1b). Here, we assessed the replicative capacity and pathogenicity of authentic BA.2.75 subvariants isolated from COVID-19 patients in established COVID-19 animal models.

## Results

### Transitions in Omicron variant prevalence throughout 2022

SARS-CoV-2 has undergone a series of variant transitions since the Omicron lineage was first observed in November of 2021. The initial global transition from Delta to the Omicron BA.1 lineage was extremely swift and was followed successively by waves of BA.2 and BA.5, with each variant, essentially replacing the previous dominant form (Fig. 1a). This may indicate that each variant has been more transmissible than the prior variant, particularly in settings with histories of prior infection and vaccination resulting in changes in immune status at the population level. BA.2.75, a BA.2 sublineage, was first detected in India in May of 2022, and since then has been increasing in sampling frequency in some parts of the world, with different BA.2.75 sublineages reaching high prevalence in different countries (Fig. 1b, Supplementary Figs. 2 and 3). As of this writing (Jan. 28, 2023), there are 95,000 BA.2.75 lineage sequences in GISAID^7,8^, and these have been sampled globally. After BA.2.75 came to high prevalence in India and Nepal, and had begun to spread to other parts of Asia (e.g., Singapore), it peaked in prevalence and was replaced by the recombinant lineage XBB (Fig. 1b). Recently, however, new BA.2.75 variant sublineages have emerged and are expanding in these countries (Fig. 1b), and these newer BA.2.75 sublineages are also increasingly sampled in many other nations (Supplementary Figs. 2 and 3 and Fig. 1b). This pattern suggests that BA.2.75 sublineages may have a selective advantage over other locally co-circulating variants, and that BA.2.75 lineage variants remain globally relevant. Currently, BA.2.75 variants coexist in a complex backdrop of newer variants including XBB (e.g., XBB.1.5) and BA.5 lineages (e.g., BQ.1.1) (Fig. 1).

### BA.2.75 infection in wild-type hamsters

To characterize BA.2.75 in vivo, we amplified three BA.2.75 clinical isolates in VeroE6/TMPRSS2 cells: hCoV-19/Japan/TY41-716/2022 (TY41-716)^9^, hCoV-19/Japan/UT-NCD1757-1N/2022 (NCD1757), and hCoV-19/Japan/UT-NCD1759-1N/2022 (NCD1759). We confirmed that the S protein of all three isolates contained the nine additional amino acid changes (i.e., K147E, W152R, F157L, I210V, G257S, D339H, G446S, N460K, and Q493 (reversion)) (Supplementary Fig. 1b) that distinguish the consensus form of BA.2.75 (https://cov.lanl.gov/components/sequence/COV/pangocommonforms.comp) from a BA.2 isolate (hCoV-19/Japan/UT-NCD1288-2N/2022; NCD1288), which carries the most common circulating form of BA.2 in Spike. However, two of the isolates (NCD1757 and NCD1759) had a D574V substitution in the subdomain (SD), in addition to the nine mutations; this and several other distinctive mutations found in other proteins are summarized in Supplementary Fig. 1b.

**Fig. 2:**
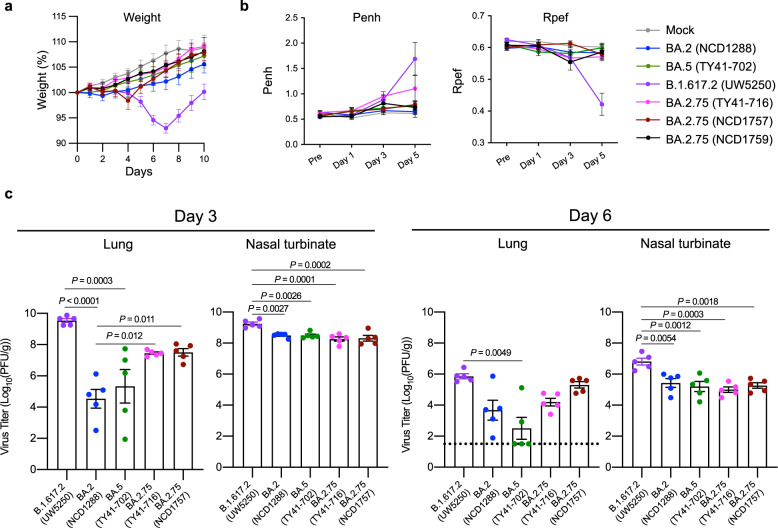
The infectivity and pathogenicity of BA.2.75 in wild-type hamsters. **a**, **b** Wild-type Syrian hamsters were intranasally inoculated with 10^5^ PFU in 30 μL of BA.2 (NCD1288) (*n* = 9), BA.5 (TY41-702) (*n* = 9), BA.2.75 (TY41-716) (*n* = 5), BA.2.75 (NCD1757) (*n* = 5), BA.2.75 (NCD1759) (*n* = 5), B.1.617.2 (UW5250) (*n* = 9), or PBS (mock) (*n* = 8). **a** Body weights of virus-infected and mock-infected hamsters were monitored daily for 10 days. Data are presented as the mean percentages of the starting weight (±s.e.m.). **b** Pulmonary function analyses in virus-infected and mock-infected hamsters. Penh and Rpef were measured by using whole-body plethysmography. Mean ± s.e.m. Data were analyzed by using a two-way ANOVA followed by Tukey’s multiple comparisons test. **c** Virus replication in infected Syrian hamsters. Hamsters (*n* = 10) were intranasally inoculated with 10^5^ PFU in 30 μL of BA.2 (NCD1288), BA.5 (TY41-702), BA.2.75 (TY41-716), BA.2.75 (NCD1757), or B.1.617.2 (UW5250) and euthanized at 3 and 6 dpi for virus titration (*n* = 5/day). Virus titers in the nasal turbinates and lungs were determined by performing plaque assays with Vero E6-TMPRSS2-T2A-ACE2 cells. Vertical bars show the mean ± s.e.m. Points indicate data from individual hamsters. The lower limit of detection is indicated by the horizontal dashed line. Data were analyzed by using a one-way ANOVA with Tukey’s multiple comparisons test (titers in the lungs at 3 dpi and nasal turbinates at 3 and 6 dpi) or the Kruskal–Wallis test followed by Dunn’s test (titers in the lungs at 6 dpi). *P* values of <0.05 were considered statistically significant. Data are from one experiment.

We first evaluated the pathogenicity of the BA.2.75 isolates in wild-type Syrian hamsters, a well-established small animal model for the study of COVID-19^10–12^. Syrian hamsters were intranasally inoculated with 10^5^ plaque-forming units (PFU) of BA.2.75 (TY41-716, NCD1757, or NCD1759). For comparison, additional hamsters were infected with clinical isolates of BA.2 (10^5^ PFU of NCD1288)^2,13^, BA.5 [10^5^ PFU of hCoV-19/Japan/TY41-702/2022 (TY41-702)]^4^, or B.1.617.2 [10^5^ PFU of hCoV-19/USA/WI-UW-5250/2021 (Delta: UW5250)]^3^. Intranasal infection with B.1.617.2 resulted in significant body weight loss by 7 days post-infection (dpi) (−7.0%) (Fig. 2a), consistent with our previous observations^3,4^. By contrast, most of the animals infected with any of the three BA.2.75 isolates gained weight over the 6-day experiment, similar to BA.2-, BA.5-, or mock-infected animals. We also examined pulmonary functions in the infected hamsters by measuring Penh and Rpef, which are surrogate markers for bronchoconstriction and airway obstruction, respectively, by using a whole-body plethysmography system. Inoculation of hamsters with the BA.2, BA.5, BA.2.75 (NCD1757), or BA2.75 (NCD1759) isolate did not cause substantial changes in either Penh or Rpef at any timepoint post-infection compared to the mock-infected group. Infection with BA.2.75 (TY41-716) caused a slight increase in Penh at 3 and 5 dpi, although no statistically significant differences in Penh values were observed among BA.2-, BA.5-, and BA.2.75 (TY41-716)-infected animals. Consistent with our previous data, infection with B.1.617.2 caused significant changes in Rpef in comparison with the five Omicron isolates (Fig. 2b).

We next assessed levels of infection in the respiratory tract of wild-type Syrian hamsters (Fig. 2c). Hamsters were intranasally infected with 10^5^ PFU of BA.2.75 (TY41-716), BA.2.75 (NCD1757), BA.2 (NCD1288), BA.5 (TY41-702), or B.1.617.2 (Delta: UW5250); at 3 and 6 dpi, the animals were sacrificed, and their nasal turbinates and lungs were collected for virus titration. The virus titers were determined by performing plaque assays on Vero E6-TMPRSS2-T2A-ACE2 cells. BA.2 (NCD1288), BA.5 (TY41-702), BA.2.75 (TY41-716), and BA.2.75 (NCD1757) replicated in the nasal turbinates of the infected animals with no significant differences in viral titers at both timepoints examined. However, the virus titers in the nasal turbinates were significantly lower in the respiratory tract of animals infected with the BA.2, BA.5, BA.2.75 (TY41-716), or BA.2.75 (NCD1757) isolates, compared to animals infected with B.1.617.2 [mean differences in viral titer = 0.75, 0.75, 0.98, or 0.94 and 1.4, 1.6, 1.8, or 1.5 log_10_ (PFU/g) at 3 and 6 dpi, respectively].

Consistent with our previous report^4^, the virus titers in the lungs of animals infected with BA.2 or BA.5 were lower than those in animals infected with B.1.617.2 [mean differences in viral titer = 5.0 or 4.2 and 2.2 or 3.4 log_10_ (PFU/g) at 3 and 6 dpi, respectively], although the difference was not statistically significant between the BA.2- and B.1.617.2-infected groups at 6 dpi. The lung titers in the BA.2.75 (TY41-716)-infected groups were also lower than those in the B.1.617.2-infected groups [mean difference in viral titer = 2.1 and 1.7 log_10_ (PFU/g) at 3 and 6 dpi, respectively], although these differences did not reach statistical significance. The viral titers in the lungs of another BA.2.75 strain (NCD1757)-infected groups were similarly lower than those in the B.1.617.2-infected group at 3 dpi [mean differences in viral titer = 2.0 log_10_ (PFU/g)]; however, animals infected with BA.2.75 or B.1.617.2 had similar titers in the lungs at 6 dpi. The lung titers in the BA.2.75 (TY41-716)- and BA.2.75 (NCD1757)-infected groups were higher than those in BA.5-infected groups [for BA.2.75 (TY41-716), mean differences in viral titer = 2.1 and 1.7 log_10_ (PFU/g), at 3 and 6 dpi, respectively; for BA.2.75 (NCD1757), mean differences in viral titer = 2.2 and 2.8 log_10_ (PFU/g), at 3 and 6 dpi, respectively]; however, the differences were not statistically significant among the three groups. At 3 dpi, the virus titers in the lungs were significantly higher in the respiratory tract of animals infected with BA.2.75 (TY41-716), compared to animals infected with BA.2 (NCD1288) [mean difference in viral titer = 2.9 log_10_ (PFU/g)]; however, at 6 dpi, similar titers were detected in the lungs of animals inoculated with BA.2.75 (TY41-716) or BA.2. The viral titers in the lungs of the BA.2.75 (NCD1757)-infected groups were also higher than those in the BA.2 (NCD1288)-infected groups [mean differences in viral titer = 3.0 and 1.6 log_10_ (PFU/g), at 3 and 6 dpi, respectively], although the difference was not statistically significant between the BA.2.75 (NCD1757)- and BA.2-infected groups at 6 dpi. Taken together, these results suggest that the replicative ability of BA.2.75 in the lungs of wild-type hamsters is higher than that of previous Omicron variants, including BA.2 and BA.5.

### Histopathological findings in the lungs of SARS-CoV-2 BA.2.75 virus-inoculated Syrian hamsters

**Fig. 3:**
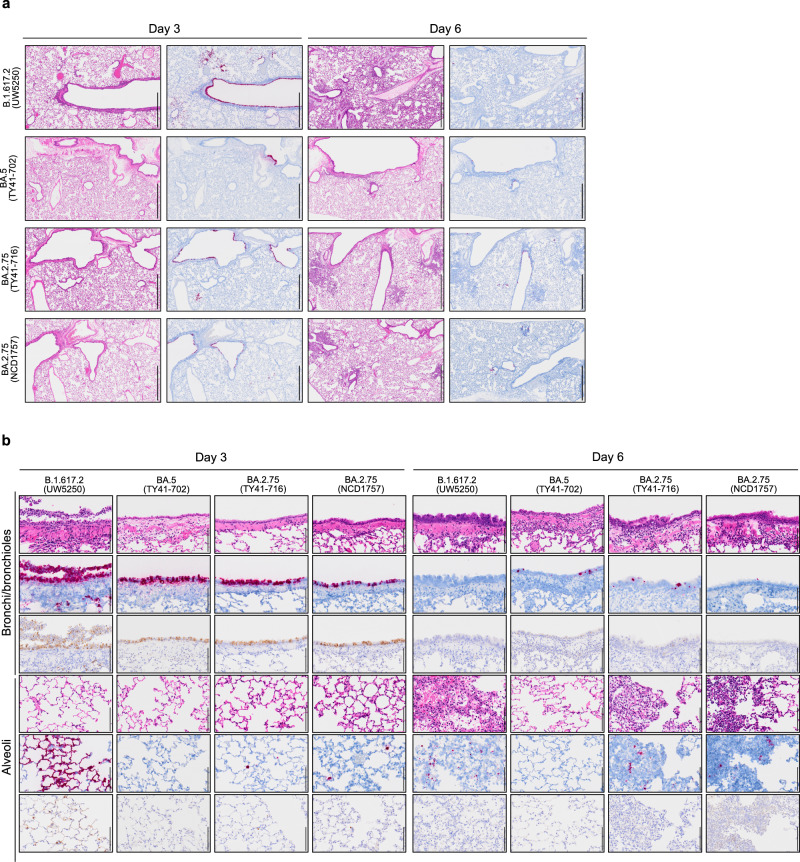
Histopathological findings in hamsters inoculated with BA.2.75. Wild-type Syrian hamsters (*n* = 5, per group) were inoculated with 10^5^ PFU of BA.2.75 (TY41-716), BA.2.75 (NCD1757), BA.5 (TY41-702), or B.1.617.2 (UW5250) and sacrificed at 3 or 6 dpi for histopathological examinations. **a** Representative images of the lungs at low magnification are shown. Left columns, hematoxylin and eosin staining. Right columns, in situ hybridization targeting the nucleocapsid gene of SARS-CoV-2. Scale bars, 1 mm. **b** Representative images of the bronchi/bronchioles and alveoli at high magnification are shown. Upper rows, hematoxylin and eosin staining. Middle rows, in situ hybridization targeting the nucleocapsid gene of SARS-CoV-2. Lower rows: immunohistochemistry for the detection of SARS-CoV-2 nucleocapsid protein by a rabbit polyclonal antibody. Scale bars, 100 µm. Data are from one experiment.

The lungs of Syrian hamsters that were inoculated with BA.2.75, BA.5, or B.1.617.2 were also analyzed histopathologically. Hamsters were intranasally inoculated with BA.2.75 (TY41-716), BA.2.75 (NCD1757), BA.5 (TY41-702), or B.1.617.2 (Delta, UW5250) and euthanized at 3 and 6 dpi for histopathological evaluation; representative images are shown in Fig. 3. This examination revealed that inflammation was not obvious in the lungs of either BA.2.75 (TY41-716)-, BA.2.75 (NCD1757)-, or BA.5-inoculated animals at 3 dpi; however, infiltration of inflammatory cells such as mononuclear cells and neutrophils was observed in peribronchial and peribronchiolar regions in these two groups at 6 dpi (Fig. 3a, b and Supplementary Fig. 4). It is noteworthy that focal pneumonia, characterized by patchy inflammation interspersed in alveolar regions, was observed in the lungs of both BA.2.75 (TY41-716 and NCD1757)-inoculated animals at 6 dpi. However, there was no obvious pneumonia in the lungs of BA.5-inoculated animals at the same timepoint. By contrast, in the lungs of the B.1.617.2-inoculated animals, peribronchial and peribronchiolar inflammation was prominent at 3 dpi, and extensive pneumonia with focal alveolar hemorrhage was observed in the alveolar regions at 6 dpi (Fig. 3a, b and Supplementary Fig. 4). In addition, we detected viral RNA and protein in the lung tissue of BA.2.75 (TY41-716)-, BA.2.75 (NCD1757)-, BA.5-, or B.1.617.2-infected hamsters by use of in situ hybridization and immunohistochemistry. These analyses revealed that viral RNA and antigen were readily detected on bronchial/bronchiolar epithelium in all BA.2.75 (TY41-716)-, BA.2.75 (NCD1757)-, and BA.5-inoculated animals at 3 dpi with a clear decrease in positive cells over time (Fig. 3a, b). In the alveolar regions, a small number of cells were positive for viral RNA or antigen in both the BA.2.75 (TY41-716)- and BA.2.75 (NCD1757)-inoculated groups at both timepoints examined, and fewer cells were positive in the BA.5-inoculated group at the corresponding timepoints (Fig. 3a, b). Comparatively, at 3 dpi, the lungs of the B.1.617.2-inoculated hamsters had diffusely positive viral RNA and antigen in the bronchial/bronchiolar areas and patchily positive viral RNA and antigen in the alveolar regions (Fig. 3a, b). Both BA.2.75 (TY41-716) and BA.2.75 (NCD1757) thus produced mild viral pneumonia in the hamster model with attenuated pathogenicity compared with B.1.617.2, whereas BA.5 did not cause obvious viral pneumonia. In addition, the numbers of viral RNA/antigen-positive cells in the alveolar regions of the BA.2.75 (TY41-716) and BA.2 75 (NCD1757)-inoculated animals were higher than those in the BA.5-inoculated animals, but lower than those in the B.1.617.2-inoculated ones.

### BA.2.75 replication in the extrapulmonary organs of wild-type hamsters

As described above, BA.2.75 differs from BA.2 by nine amino acids in the Spike; however, two (NCD1757 and NCD1759) of the three BA.2.75 strains we tested have a substitution, in addition to the nine substitutions (Supplementary Fig. 1b). For the following experiments, we therefore characterized only the TY41-716 strain, as a representative of the most common circulating form of BA.2.75 with respect to Spike.

Previously, we and other groups have reported that ancestral SARS-CoV-2 is detectable in the brain, liver, spleen, heart, and kidneys of wild-type hamsters^10,11^. To examine whether BA.2.75 replicates in the extrapulmonary organs of wild-type hamsters, four hamsters per group were intranasally infected with 10^5^ PFU of BA.2.75 (TY41-716), BA.5 (TY41-702), or B.1.617.2 (Delta: UW5250); at 5 dpi, the heart, kidneys, spleen, brain, and lungs of the infected animals were collected to measure infectious virus titers. Subgenomic viral RNAs in these organs also were examined by using quantitative RT-PCR (qRT-PCR). Both viral RNA levels and viral titers in the lungs of BA.2.75-infected hamsters were significantly higher than those in the lungs of BA.5-infected hamsters and significantly lower than those in the lungs of B.1.617.2-infected hamsters at 5 dpi, consistent with our observations in wild-type hamsters infected with these viruses at days 3 and 6 after infection (Supplementary Fig. 5). No infectious virus was detected from any of the extrapulmonary organs of the BA.5- or BA.2.75-infected animals (Supplementary Fig. 5a). In contrast, virus was recovered from the heart of two of the four B.1.617.2-infected animals and the kidney of one of these four animals. No viral RNA was detected in the extrapulmonary organs of almost all of BA.5- or BA.2.75-infected animals, with the exception of the brain of one of the four animals infected with BA.2.75 and the kidney of one of the four animals infected with BA.2.75 (Supplementary Fig. 5b). Viral RNA was detected in the heart of all four B.1.617.2-infected animals and the kidney of one of the four animals (Supplementary Fig. 5b). These results suggest that BA.2.75 mainly replicates in the respiratory organs of wild-type hamsters.

### Host responses in BA.2.75-infected wild-type hamsters

To evaluate the host responses after BA.2.75 infection, we measured several proinflammatory and innate cytokine gene expression levels in the lungs of hamsters at 3 dpi by using qRT-PCR. The expression levels of all four genes [i.e., *Interleukin (Il)1β, Il6*, *tumor necrosis factor (Tnf)α*, and *interferon (Ifn)γ*] tested were remarkably lower in the lungs of BA.2, BA.5-, or BA.2.75-infected animals than in those of B.1.617.2-infected animals, although no statistically significant differences in the expression levels of the *Tnfα* and *Ifnγ* genes were found between the BA.2.75-infected and B.1.617.2-infected groups. Among the three Omicron variant groups, the expression levels of all four genes in the lungs were slightly higher in the BA.2.75-infected group than in the BA.2- or BA.5-infected group, although these difference did not reach statistical significance (Supplementary Fig. 6) [for *Il1β*, mean fold-changes relative to Naive were 1.11, 0.97, and 0.88, respectively; for *Il6*, mean fold-changes relative to Naive were 1.78, 1.47, and 0.92, respectively; for *Tnfα*, mean fold-changes relative to Naive were 2.19, 1.53, and 1.83, respectively; and for *Ifnγ*, mean fold-changes relative to Naïve were 3.52, 1.39, and 1.27, respectively]. These results suggest that infection with BA.2.75 may induce higher proinflammatory responses in the lungs of infected hamsters compared with BA.2 or BA.5 infections, but the responses are attenuated compared to those in response to B.1.617.2 infection, which is consistent with the results of histopathologic examination and virus replication in the lungs of wild-type hamsters.

### BA.2.75 infection in hACE2-expressing hamsters

**Fig. 4:**
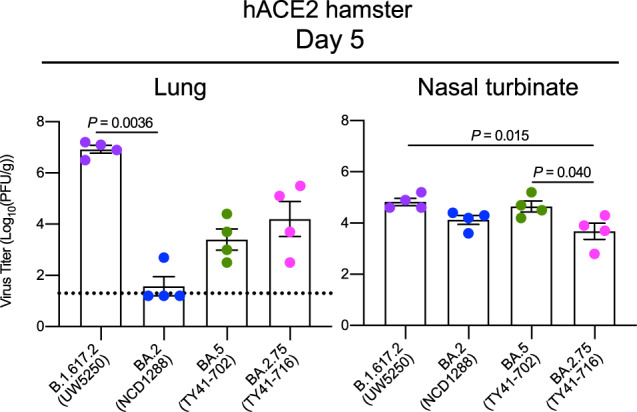
The infectivity of BA.2.75 in hACE2-expressing hamsters. hACE2-expressing Syrian hamsters (*n* = 4) were intranasally inoculated with 10^5^ PFU in 30 μL of BA.2 (NCD1288), BA.5 (TY41-702), BA.2.75 (TY41-716), or B.1.617.2 (UW5250). Infected animals were euthanized at 5 dpi for virus titration (*n* = 4/group). Virus titers in the nasal turbinates and lungs were determined by performing plaque assays with Vero E6-TMPRSS2-T2A-ACE2 cells. Vertical bars show the mean ± s.e.m. Points indicate data from individual animals. The lower limit of detection is indicated by the horizontal dashed line. Data were analyzed by using a one-way ANOVA with Tukey’s multiple comparisons test (titers in the nasal turbinates) or the Kruskal–Wallis test followed by Dunn’s test (titers in the lungs). *P* values of <0.05 were considered statistically significant. Data are from one experiment.

We investigated the infectivity of BA.2.75 in respiratory organs by using a more susceptible model, specifically transgenic hamsters expressing hACE2 (Fig. 4). At 5 dpi, the virus titers in the lungs and nasal turbinates of hACE2-expressing hamsters infected with BA.2.75 (TY41-716) were lower than those in animals infected with B.1.617 (UW5250) [mean differences in viral titer = 2.7 and 1.1 log_10_ (PFU/g), respectively], although the differences in the lungs were not statistically significant between the two groups. Similar titers were detected in the lungs of animals inoculated with BA.2.75 (TY41-716) or BA.5 (TY41-702); however, the virus titers in the nasal turbinates of the animals infected with BA.2.75 were slightly but significantly lower than in those infected with BA.5 [mean differences in viral titer = 0.98 log_10_ (PFU/g)]. The virus titers in the lungs were substantially higher in the respiratory tract of animals infected with BA.2.75 (TY41-716) compared with animals infected with BA.2 (NCD1288) [mean differences in viral titer = 2.6 log_10_ (PFU/g)], although animals infected with BA.2.75 or BA.2 exhibited similar viral titers in nasal turbinates. These results suggest that BA.2.75 may have a higher replicative ability than BA.2 in the lungs of hACE2 transgenic hamsters.

### The replicative fitness of BA.2.75 compared with that of BA.5 in wild-type hamsters

To further investigate the replicative fitness of BA.2.75, we compared the growth of BA.2.75 in wild-type hamsters with that of BA.5, which is currently the dominant variant circulating globally. Wild-type hamsters were intranasally inoculated with 2 × 10^5^ PFU of a mixture of BA.2.75 (TY41-716) and BA.5 (TY41-702) at ratios of 1:1, 1:3, 1:19, or 1:199. At 4 dpi, the proportion of each virus in the nasal turbinates and lungs of the infected hamsters was determined by using next-generation sequencing (NGS). The proportion was calculated on the basis of the differences between these two viruses across 6 regions in the S protein.

**Fig. 5:**
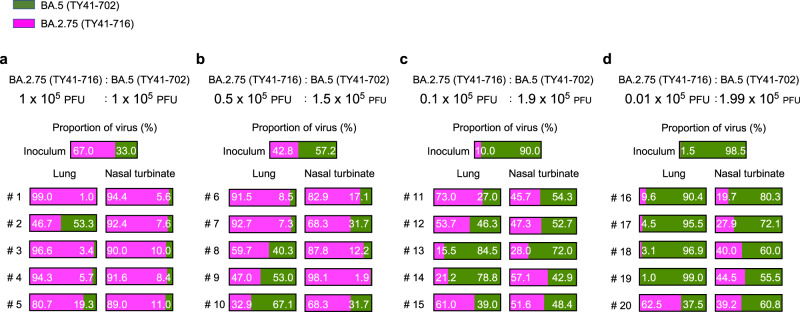
Relative viral fitness of BA.2.75 and BA.5 in wild-type hamsters. BA.2.75 (TY41-716) and BA.5 (TY41-702) were mixed at a 1:1 (**a**), 1:3 (**b**), 1:19 (**c**), or 1:199 (**d**) ratio on the basis of their infectious titers, and the virus mixture (total 2 × 10^5^ PFU in 60 µL) was intranasally inoculated into wild-type hamsters (*n* = 5). Nasal turbinates and lungs were collected from the infected animals at 4 dpi and analyzed using next-generation sequencing (NGS). Shown are the relative proportions of BA.5 and BA.2.75 in the infected wild-type animals. Data are from one experiment.

NGS analysis revealed that the proportion of BA.2.75 had increased in the nasal turbinates and lungs of all infected animals compared to that in each inoculum for any ratio, except for the lung samples from hamsters 2, 10, and 19 (Fig. 5). For animals inoculated with a 1:1 or 1:3 ratio of BA.2.75:BA.5, the lung and nasal turbinate samples showed a greater proportion of BA.2.75, except for the lung sample from hamsters 2, 9, and 10 (Fig. 5a, b). Of note, even though the proportion of BA.2.75 in the inoculum was much lower than that of BA.5 (i.e., a 1:19 or 1:199 mixture of BA.2.75:BA.5), BA.2.75 became dominant in the lungs of four (#s 11, 12, 15, and 20) of the ten animals (Fig. 5c, d). Taken together, these results suggest that BA.2.75 may have greater replicative fitness than BA.5, especially in the upper respiratory tract.

## Discussion

We previously showed that Omicron sublineage BA.2 and BA.5 variants exhibit similar pathogenicity in rodent models by using several clinical isolates, and showed that both variants are significantly attenuated compared to previous circulating VOCs^2,4^. Here, we evaluated the replication and pathogenicity of Omicron sublineage BA.2.75 variants in hamsters. Our data show that there are no substantial differences in weight change among hamsters infected with BA.2.75, BA.2, or BA.5 (Fig. 2a); however, viral titers in the lungs of BA.2.75-infected hamsters were higher than those in the lungs of BA.2- or BA.5-infected hamsters (Fig. 2c). In addition, in competition assays, BA.2.75 replicated better than BA.5 in the lungs (Fig. 5). Of note, in the lungs of BA.2.75-inoculated hamsters, we observed focal pneumonia, characterized by patchy inflammation interspersed in alveolar regions, indicating that BA.2.75 can cause mild pneumonia (Fig. 3 and Supplementary Fig. 4). In contrast, BA.5 mainly affected the bronchi, resulting in bronchitis/bronchiolitis, and did not cause obvious pneumonia (Fig. 3). Similar results were observed with hamsters infected with BA.1, BA.2, or BA.4^2–4^. These findings suggest that among the Omicron variants, the Omicron subvariant BA.2.75 causes the most severe tissue damage in the lungs of hamsters.

Omicron variants, including BA.1 or BA.2, are less likely than Delta variants to be associated with pneumonia in COVID-19 patients^14–16^, consistent with our previous data obtained in a hamster model^2,3^. However, in the present study, we found that BA.2.75 can cause focal viral pneumonia in hamsters, unlike the other Omicron variants (i.e., BA.1, BA.2, BA.4, and BA.5)^2–4^. The reason for this is unclear; however, it might be due to differences in the binding affinity of the S protein for hACE2 among BA.1, BA.2, BA.4, BA.5, and BA.2.75. Recent studies have reported that the RBD of BA.2.75 exhibits higher binding affinity for the hACE2 receptor than that of BA.2 and BA.4/5^5,6^. SARS-CoV-2 enters cells in two distinct ways: by fusion of the viral lipid envelope with the target cell plasma membrane or fusion of the viral envelope with the endosomal membrane after internalization through the endocytic pathway^17–19^. The internalization of SARS-CoV-2 via the endocytic pathway is believed to be induced by the binding of the virus to ACE2^20,21^. The Omicron variants have been shown to preferentially utilize the endocytic pathway to enter cells^22,23^. In addition, previous studies have demonstrated that the enhanced binding affinity between ACE2 and the RBD increases the efficiency of SARS-CoV-2 entry^24,25^. Therefore, the higher ACE2 binding affinity of BA.2.75 may enhance its ability to infect the lungs, thereby allowing BA.2.75 to cause viral pneumonia in hamsters. Also, this higher ACE2 affinity of BA.2.75 may increase its competitive fitness compared to BA.5 in the respiratory tracts of hamsters, as observed in our in vivo competition assay (Fig. 5). Further investigations are required to understand the underlying mechanism of the increased fitness of BA.2.75, for example, the efficiency of virus replication including virus entry, viral RNA synthesis, and budding, and the ability to escape from local immune responses.

We note two key limitations in this study: (1) although hamsters are one of the most widely used animals that are known to be susceptible to SARS-CoV-2, including mice and non-human primates^10–12^, it is unclear whether the BA.2.75 variant causes more clinically severe respiratory disease than other Omicron variants in humans; and (2) our study was performed in immunologically naïve animals; however, many people have already acquired immunity to SARS-CoV-2 through natural infection and/or vaccination. Therefore, it remains unclear whether our data reflect the clinical outcome in patients with immunity against SARS-CoV-2. Clinical studies are needed to corroborate our findings in the hamster model.

In summary, our data show that, compared to BA.5 and BA.2, BA.2.75 can replicate efficiently in the lungs of hamsters and cause more severe respiratory disease. This higher replicative ability of BA.2.75 in the lower respiratory tract may affect the clinical outcome in infected humans.

## Methods

### Variant tracking strategies

Figure 1, Supplementary Figs. 2, and 3 show transitions between variant forms, emphasizing the continuing expansion of the BA.2.75 variant sublineages that is indicative of a possible selective advantage over co-circulating forms in some geographic regions. Web-based updates of these figures based on the most recent GISAID data can be generated via the “Embers” and “Isotonic Regression” tools at the Los Alamos National Laboratory SARS-CoV-2 variant analysis website (https://cov.lanl.gov); the methods used here were first developed by Korber et al.^26^. To resolve the lineage groupings shown in Fig. 1, we first aligned all sequences in GISAID, and then defined all distinct forms of the spike protein in the GISAID sampled within the last two months. This provides a contemporary view of currently circulating common forms. These variant forms were then tallied according to their assigned Pango lineage designations (https://cov.lanl.gov/components/sequence/COV/pangocommonforms.comp); often distinct Pango lineages share the precise form of the Spike protein. We next grouped Pango lineages that share the same baseline Spike for tracking purposes, and this enabled us to resolve five major distinctive sublineages of BA.2.75 (Supplementary Fig. 1), with a sixth category of “others”, which includes both more ancestral and rarer forms. Figure 1 was created using Embers based on those Pango lineage groupings, and displays running weekly counts and proportions of variants at different geographic levels.

The Isotonic Regression analysis used for Supplementary Figs. 2 and 3 explores the dynamics of the transition towards higher frequencies of BA.2.75 sublineages over time, and can be run through the cov.lanl.gov Isotonic Regression tool (http://cov-dev.lanl.gov/content/sequence/ISORG/isorg.html). All countries globally that had a given BA.2.75 sublineage that was well enough established to have been sampled ten or more times are included in the analysis. A one-sided resampling statistic is used to evaluate whether increased sampling over time of a given sublineages is significant^7,8,26^. This was used to identify nations where BA.2.75 sublineages are currently common and increasingly sampled for inclusion in Fig. 1b. The full Isotonic Regression analysis of the two most common sublineages of BA.2.75, CH.1, and BN.1, are shown in Supplementary Figs. 2 and 3.

The sequence data sets for these analyses and figures were uploaded from GISAID 2023-01-27^7,8^. We are grateful for the global contributions of the scientific community to GISAID to enable this work, and the following is a summary of the data that was provided: GISAID Identifier: EPI_SET_230129sh (10.55876/gis8.230129sh, https://epicov.org/epi3/epi_set/230129sh?main=true). All genome sequences and associated metadata including the contributors of each individual sequence with details such as accession number, virus name, collection date, originating lab, submitting lab, and the list of authors in this dataset are published in GISAID’s EpiCoV database. EPI_SET_230129sh comprises 6,947,638 individual genome sequences. The collection dates range from Jan 1, 2022 to Jan 25, 2023 and data were collected in 205 countries and territories, but the sampling after Jan 18, 2023 is sparse so those data are excluded from the analysis.

### Cells

VeroE6/TMPRSS2 (JCRB 1819) cells^27^ were propagated in the presence of 1 mg/ml geneticin (G418; Invivogen) and 5 μg/ml plasmocin prophylactic (Invivogen) in Dulbecco’s modified Eagle’s medium (DMEM) containing 10% Fetal Calf Serum (FCS). Vero E6-TMPRSS2-T2A-ACE2 cells (provided by Dr. Barney Graham, NIAID Vaccine Research Center, available at BEI Resources, NR-54970) were cultured in DMEM supplemented with 10% FCS, 100 U/mL penicillin–streptomycin, and 10 μg/mL puromycin. VeroE6/TMPRSS2 and Vero E6-TMPRSS2-T2A-ACE2 cells were maintained at 37 °C with 5% CO_2_. The cells were regularly tested for mycoplasma contamination by using PCR, and confirmed to be mycoplasma-free.

### Viruses

hCoV-19/Japan/UT-NCD1288-2N/2022 (BA.2; NCD1288)^2,13^, hCoV-19/Japan/TY41-716/2022 (BA.2.75; TY41-716)^9^, hCoV-19/Japan/UT-NCD1757-1N/2022 (BA.2.75; NCD1757), hCoV-19/Japan/UT-NCD1759-1N/2022 (BA.2.75; NCD1759), hCoV-19/Japan/TY41-702/2022 (BA.5; TY41-702)^4^, and hCoV-19/USA/WI-UW-5250/2021 (B.1.617.2; UW5250)^3,28^ were propagated in VeroE6/TMPRSS2 cells in VP-SFM (Thermo Fisher Scientific). BA.2.75 (NCD1757) and BA.2.75 (NCD1759) were subjected to next-generation sequencing (NGS) (see “Whole-genome sequencing”). All experiments with SARS-CoV-2 were performed in enhanced biosafety level 3 (BSL3) containment laboratories at the University of Tokyo and the National Institute of Infectious Diseases, Japan, which are approved for such use by the Ministry of Agriculture, Forestry, and Fisheries, Japan, or in BSL3 agriculture containment laboratories at the University of Wisconsin-Madison, which are approved for such use by the Centers for Disease Control and Prevention and by the US Department of Agriculture.

### Animal experiments and approvals

Animal studies were carried out in accordance with the recommendations in the Guide for the Care and Use of Laboratory Animals of the National Institutes of Health. The protocols were approved by the Animal Experiment Committee of the Institute of Medical Science, the University of Tokyo (approval number PA19-75) and the Institutional Animal Care and Use Committee at the University of Wisconsin, Madison (assurance number V006426). Virus inoculations were performed under isoflurane, and all efforts were made to minimize animal suffering. In vivo studies were not blinded, and animals were randomly assigned to infection groups. No sample-size calculations were performed to power each study. Instead, sample sizes were determined based on prior in vivo virus challenge experiments.

### Experimental infection of Syrian hamsters

Under isoflurane anesthesia, five to nine 6-week-old male wild-type Syrian hamsters (Japan SLC Inc., Shizuoka, Japan) per group were intranasally inoculated with 10^5^ PFU (in 30 μL) of BA.2 (NCD1288), BA.2.75 (TY41-716), BA.2.75 (NCD1757), BA.2.75 (NCD1759), BA.5 (TY41-702), or B.1.617.2 (UW5250). Baseline body weights were measured before infection. Body weight was monitored daily for 10 days. For virological and pathological examinations, ten hamsters per group were intranasally infected with 10^5^ PFU (in 30 μL) of BA.2 (NCD1288), BA.2.75 (TY41-716), BA.2.75 (NCD1757), BA.5(TY41-702), or B.1.617.2 (UW5250); 3 and 6 dpi, five animals were euthanized and nasal turbinates and lungs were collected. The virus titers in the nasal turbinates and lungs were determined by use of plaque assays on Vero E6-TMPRSS2-T2A-ACE2 cells.

To investigate the replication of SARS-CoV-2 variants in the extrapulmonary organs of wild-type hamsters, four six-week-old female wild-type Syrian hamsters (Envigo, Indianapolis, IN, USA) per group were intranasally inoculated with 10^5^ PFU (in 30 μL) of BA.2.75 (TY41-716), BA.5 (TY41-702), or B.1.617.2 (UW5250). At 5 dpi, four animals were euthanized and lungs, heart, kidneys, spleen, and brain were collected. The virus titers or levels of subgenomic RNA (sgRNA) targeting the N gene in these organs were determined by use of plaque assays on Vero E6-TMPRSS2-T2A-ACE2 cells or quantitative reverse transcription PCR (qRT-PCR) (see below), respectively.

For co-infection studies, BA.2.75 (TY41-716) was mixed with BA.5 (TY41-702) at a 1:1, 1:3, 1:19, or 1:199 ratio on the basis of their titers, and each virus mixture (total 2 × 10^5^ PFU in 60 µL) was inoculated into five six-week-old male wild-type hamsters. At 4 dpi, five animals were euthanized and nasal turbinates and lungs were collected to determine virus titers.

The K18-hACE2 transgenic hamster line (line M41) were developed by using a piggyBac-mediated transgenic approach. The K18-hACE2 cassette from the pK18-hACE2 plasmid was transferred into a piggyBac vector, pmhyGENIE-3, for pronuclear injection^29^. Then, female 6–8-week-old K18-hACE2 homozygous transgenic hamsters, whose hACE2 expression was confirmed, were intranasally inoculated with 10^5^ PFU (in 30 μL) of BA.2 (NCD1288), BA.5 (TY41-702), BA.2.75 (TY41-716), or B.1.617.2 (UW5250). At 5 dpi, the animals were euthanized and nasal turbinates and lungs were collected. The virus titers in the nasal turbinates and lungs were determined by use of plaque assays on Vero E6-TMPRSS2-T2A-ACE2 cells.

### Lung function

Respiratory parameters were measured by using a whole-body plethysmography system (PrimeBioscience) according to the manufacturer’s instructions. In brief, infected hamsters were placed in the unrestrained plethysmography chambers and allowed to acclimate for 1 min before data were acquired over a 3-min period by using FinePointe software.

### Histopathology

Histopathological examination was performed as previously described^2–4^. Excised animal lungs were fixed in 4% paraformaldehyde in phosphate-buffered saline (PBS) and processed for paraffin embedding. The paraffin blocks were sliced into 3-µm-thick sections and mounted on silane-coated glass slides, followed by hematoxylin and eosin (H&E) stain for histopathological examination. To detect SARS-CoV-2 RNA, in situ hybridization was performed using an RNA scope 2.5 HD Red Detection kit (Advanced Cell Diagnostics, Newark, California) with an antisense probe targeting the nucleocapsid gene of SARS-CoV-2 (Advanced Cell Diagnostics) following manufacturer’s instructions. Tissue sections were also processed for immunohistochemistry with a rabbit polyclonal antibody for SARS-CoV nucleocapsid protein (ProSpec; ANT-180, 1:500 dilution, Rehovot, Israel), which cross-reacts with SARS-CoV-2 nucleocapsid protein. Specific antigen-antibody reactions were visualized by means of 3,3′-diaminobenzidine tetrahydrochloride staining using the Dako Envision system (Dako Cytomation; K4001, 1:1 dilution, Glostrup, Denmark).

### Whole-genome sequencing

Viral RNA was extracted by using a QIAamp Viral RNA Mini Kit (QIAGEN). The whole genome of SARS-CoV-2 was amplified by using a modified ARTIC network protocol in which some primers were replaced or added^30,31^. Briefly, viral cDNA was synthesized from the extracted RNA by using a LunarScript RT SuperMix Kit (New England BioLabs). The DNA was then amplified by performing a multiplexed PCR in two pools using the ARTIC-N5 primers and the Q5 Hot Start DNA polymerase (New England BioLabs)^32^. The DNA libraries for Illumina NGS were prepared from pooled amplicons by using a QIAseq FX DNA Library Kit (QIAGEN) and were then analyzed by using the iSeq 100 System (Illumina). To determine the sequences of BA.2.75 (NCD1757) and BA.2.75 (NCD1759), the reads were assembled by the CLC Genomics Workbench (version 22, Qiagen) with the Wuhan/Hu-1/2019 sequence (GenBank accession no. MN908947) as a reference. The sequences of BA.2.75 (NCD1757) and BA.2.75 (NCD1759) were deposited in Genbank with accession IDs: OQ326841 and OQ326844, respectively. For the analysis of the ratio of BA.5 to BA.2.75 after co-infection, the ratio of BA.2.75 to BA.5 was calculated from the 6 amino acid differences in the S gene between the two viruses. Samples with more than 300 read-depths were analyzed.

### Quantitative reverse transcription PCR (qRT-PCR)

For detection of subgenomic viral RNA (sgRNA), RNA extraction was carried out with an QIAamp Viral RNA Mini Kit (Qiagen). Approximately 400–600 ng of total RNA including sgRNA was reverse-transcribed into cDNA by using a QuantiTect Reverse Transcription Kit (Qiagen) with oligo dT primer. The cDNA equivalent to 10 ng of input RNA was amplified and analyzed by using PowerUp SYBR Green Master Mix (Life Technologies) on QuantStudio 6 Flex (Applied Biosystems) following the manufacturer’s protocol. The primer sequences targeting sgRNA were as follows: 5′-CGGTGAACCAAGACGCAGTA-3′ (reverse primer targeting SARS-CoV-2 Nucleocapsid gene^33^). The results are presented as the mean cycle threshold (Ct) value technically performed in triplicate.

To examine innate and proinflammatory gene expression, RNA extraction was carried out with an RNeasy Mini Kit (Qiagen). Approximately 100 ng of total RNA was reacted by using a One Step TB Green PrimeScript PLUS RT-PCR Kit (Perfect Real Time) (Takara) on a LightCycler® 96 System (Roche) following the manufacturer’s protocol. The 2^−ΔΔCt^ method was used to calculate the relative mRNA expression results. The primer sequences were as follows based on a previous study^34^: *Bact*-F: 5′-ACTGCCGCATCCTCTTCCT-3′, *Bact*-R: 5′-TCGTTGCCAATGGTGATGAC-3′, *Il1β*-F: 5′-GGCTGATGCTCCCATTCG-3′, *Il1β*-R: 5′-CACGAGGCATTTCTGTTGTTCA-3′, *Il6*-F: 5′-CCTGAAAGCACTTGAAGAATTCC-3′, *Il6*-R: 5′-GGTATGCTAAGGCACAGCACACT-3′, *Tnfα*-F: 5′-GGAGTGGCTGAGCCATCGT-3′, *Tnfα*-R: 5′-AGCTGGTTGTCTTTGAGAGACATG-3′, *Ifnγ*-F: 5′-GGCCATCCAGAGGAGCATAG-3′, *Ifnγ*-R: 5′-TTTCTCCATGCTGCTGTTGAA-3′.

### Statistical analysis

GraphPad Prism software was used to analyze the data. Statistical analysis included the Kruskal–Wallis test followed by Dunn’s test, and an ANOVA with post hoc tests. Differences among groups were considered significant for *P* values <0.05.

### Reporting summary

Further information on research design is available in the Nature Portfolio Reporting Summary linked to this article.

## Supplementary information

Supplementary Information

Reporting Summary

## Data Availability

All data supporting the findings of this study are available in the paper. There are no restrictions in obtaining access to primary data. All data generated in this study are provided in the Supplementary Information/Source data file. The sequences of BA.2.75 (NCD1757) and BA.2.75 (NCD1759) determined in this study have been deposited in Genbank with accession IDs: OQ326841 and OQ326844, respectively. Source data are provided with this paper.

## Source data

Source Data

## Materials availability

All reagents described in this paper are available through Material Transfer Agreements.

## Code availability

No novel code was used in the course of the data acquisition or analysis, the most representative forms of each virus, dynamics plots, and isotonic regression analyses are available at https://cov.lanl.gov/content/index.
