# Supplementary material for: Characterization of SARS-CoV-2 Omicron BA.2.75 clinical isolates
Source: Nat Commun. 2023 Mar 23;14:1620. doi: 10.1038/s41467-023-37059-x (PMC10035475; doi:10.1038/s41467-023-37059-x)
Supplement: Supplementary file 1 — Supplementary Information [file 41467_2023_37059_MOESM1_ESM.pdf]

# Supplementary Information

### Characterization of SARS-CoV-2 Omicron BA.2.75 clinical isolates

Ryuta Uraki[1,2,14], Shun Iida[3,14], Peter J. Halfmann[4,14], Seiya Yamayoshi[1,2,14], Yuichiro Hirata[3], Kiyoko Iwatsuki-Horimoto[1], Maki Kiso[1], Mutsumi Ito[1], Yuri Furusawa[1,2], Hiroshi Ueki[1,2], Yuko Sakai-Tagawa[1], Makoto Kuroda[4], Tadashi Maemura[4], Taksoo Kim[4], Sohtaro Mine[3], Noriko Iwamoto[5], Rong Li[6], Yanan Liu[6], Deanna Larson[6], Shuetsu Fukushi[7], Shinji Watanabe[8], Ken Maeda[9], Zhongde Wang[6], Norio Ohmagari[5], James Theiler[10], Will Fischer[11,12], Bette Korber[11,12]¶, Masaki Imai[1,2]¶, Tadaki Suzuki[3]¶, and Yoshihiro Kawaoka[1,2,4,13]¶

[1]*Division of Virology, Institute of Medical Science, University of Tokyo, Tokyo 108-8639, Japan*
[2]*The Research Center for Global Viral Diseases, National Center for Global Health and Medicine Research Institute, Tokyo 162-8655, Japan*
[3]*Department of Pathology, National Institute of Infectious Diseases, Tokyo 162-8640, Japan*
[4]*Influenza Research Institute, Department of Pathobiological Sciences, School of Veterinary Medicine, University of Wisconsin-Madison, Madison, WI 53711, USA*
[5]*Disease Control and Prevention Center, National Center for Global Health and Medicine Hospital, Tokyo 162-8655, Japan*
[6]*Department of Animal, Dairy, and Veterinary Sciences, College of Agriculture and Applied Sciences, Utah State University, Logan, UT 84322, USA*
[7]*Department of Virology 1, National Institute of Infectious Diseases, Musashimurayama, Tokyo 208-0011, Japan*
[8]*Center for Influenza and Respiratory Virus Research, National Institute of Infectious Diseases, Musashimurayama, Tokyo 208-0011, Japan*
[9]*Department of Veterinary Science, National Institute of Infectious Diseases, Tokyo 162-8640, Japan*
[10]*Space Data Science and Systems, Los Alamos National Laboratory, Los Alamos, NM 87545, USA*
[11]*Theoretical Biology and Biophysics, Los Alamos National Laboratory, Los Alamos, NM 87545, USA*
[12]*New Mexico Consortium, Los Alamos, NM 87545, USA*
[13]*The University of Tokyo, Pandemic Preparedness, Infection and Advanced Research Center, Tokyo 162-8655, Japan*
[14]*These authors contributed equally*
¶*Corresponding authors*

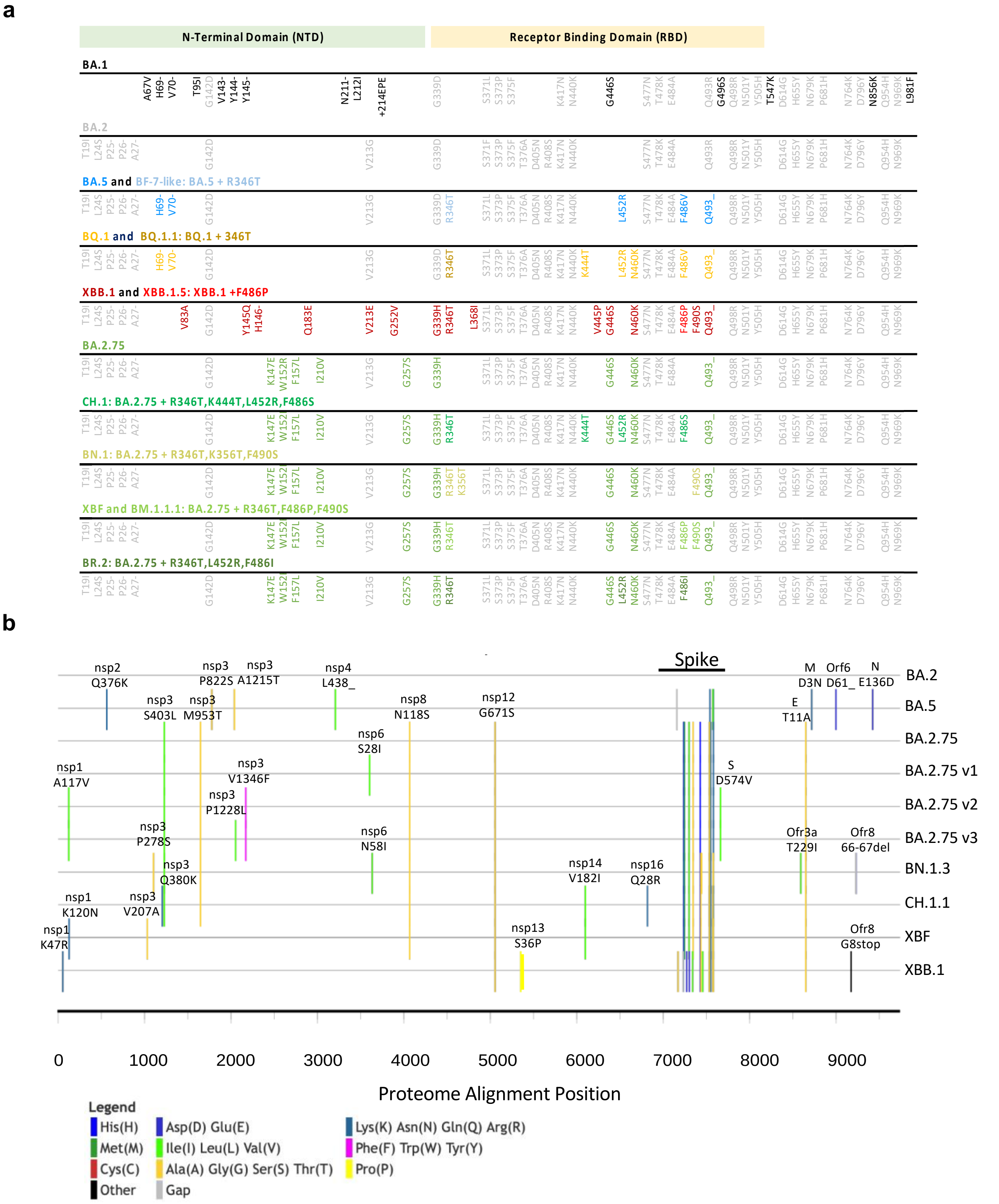


**Supplementary Figure 1. Amino acid differences between representative forms of recently emerged Omicron variants.**

a, Amino acid differences in the Spike of commonly circulating Omicron variants. BA.1 was rapidly globally replaced by BA.2; the most common form of BA.2 serves as the reference. Spike amino acid differences between the ancestral strain WIV04/2019 (Pango lineage A; Accession ID: EPI_ISL_402124 (https://gisaid.org/wiv04/))

and the baseline form of BA.2 are shown in grey. When other Omicron variants share spike BA.2-defining mutations in a given position, they are noted in grey. When they differ, the amino acid change is highlighted in the color assigned to each variant (the same color as used in **Figure 1**). Representative forms of the variants that are currently commonly circulating are shown: BA.5, BF-7-like lineages that resemble BA.5 lineages with the addition of R346T, and BQ.1 and BQ.1.1 sublineages; XBB sublineages including XBB.1.5; and several common BA.2.75 sublineages. Four representative forms of currently circulating BA.2.75 sublineage spikes are highlighted in shades of green. The recombinant lineage XBF is included here because it carries a BA.2.75 lineage Spike. Deletions are indicated by a dash (-), insertions by a plus sign (e.g., +214EPE means a three amino acid insertion of EPE after position 214). Reversions from BA.2 to the ancestral form are indicated by an underscore (_). b, Highlighting amino acid differences between BA.2, BA.5, and BA.2.75 and examples of BA.2.75 variants throughout the full proteome. Only amino differences from the most representative form of BA.2 are shown, illustrated as a tick mark. The grey line represents the full proteome. All changes in the most common forms of BA.5 and BA.2.75 relative to BA.2 are noted, as these are candidates for contributing to a selective advantage for BA.5 over BA.2, and for BA.2.75 over BA.5 and BA.2. Details for the amino acid differences are shown in part a. Amino acids that are distinctive in the three BA.2.75 variants studied in this paper are highlighted, BA.2.75 V1–V3, as well as several sublineages of BA.2.75 that have persisted into 2023. Full-length representative forms of Pango lineages are defined as the most common circulating form of a given Pango lineage.

| Countries | Number of CH.1 sequences | Number of other sequences | Total number of seuqences | CH.1/Total (%) | Number of days sampled | One sided p-value, CH.1.1 increasing |
|---|---|---|---|---|---|---|
| Australia | 671 | 19039 | 19710 | 3.4 | 82 | 0.00249 |
| Austria | 373 | 30546 | 30919 | 1.21 | 82 | 0.00249 |
| Belgium | 94 | 3676 | 3770 | 2.49 | 81 | 0.00249 |
| Brunei | 15 | 755 | 770 | 1.95 | 61 | 0.00249 |
| Cambodia | 20 | 176 | 196 | 10.2 | 54 | 0.00249 |
| Canada | 215 | 32532 | 32747 | 0.66 | 83 | 0.00249 |
| Chile | 18 | 2908 | 2926 | 0.62 | 80 | 0.00498 |
| Czech Republic | 12 | 1123 | 1135 | 1.06 | 71 | 0.00249 |
| Denmark | 1200 | 32196 | 33396 | 3.59 | 83 | 0.00249 |
| Finland | 33 | 2571 | 2604 | 1.27 | 73 | 0.00249 |
| France | 172 | 24834 | 25006 | 0.69 | 76 | 0.00249 |
| Germany | 426 | 38287 | 38713 | 1.1 | 80 | 0.00249 |
| Greece | 77 | 2977 | 3054 | 2.52 | 56 | 0.04478 |
| Hong-Kong | 139 | 576 | 715 | 19.44 | 72 | 0.00249 |
| Iceland | 51 | 1130 | 1181 | 4.32 | 81 | 0.00249 |
| India | 27 | 1362 | 1389 | 1.94 | 76 | 0.00498 |
| Indonesia | 39 | 5153 | 5192 | 0.75 | 77 | 0.00249 |
| Ireland | 618 | 4652 | 5270 | 11.73 | 85 | 0.00249 |
| Israel | 121 | 5533 | 5654 | 2.14 | 64 | 0.00249 |
| Italy | 92 | 9976 | 10068 | 0.91 | 83 | 0.00249 |
| Japan | 704 | 65446 | 66150 | 1.06 | 87 | 0.00249 |
| Liechtenstein | 13 | 137 | 150 | 8.67 | 55 | 0.99502 |
| Lithuania | 28 | 499 | 527 | 5.31 | 35 | 0.00249 |
| Luxembourg | 64 | 3673 | 3737 | 1.71 | 79 | 0.00249 |
| Malaysia | 94 | 3633 | 3727 | 2.52 | 79 | 0.00249 |
| Netherlands | 376 | 8666 | 9042 | 4.16 | 80 | 0.00249 |
| New Zealand | 726 | 4245 | 4971 | 14.6 | 53 | 0.00249 |
| Norway | 15 | 1232 | 1247 | 1.2 | 70 | 0.26617 |
| Peru | 55 | 4731 | 4786 | 1.15 | 63 | 0.00249 |
| Poland | 17 | 810 | 827 | 2.06 | 74 | 0.00498 |
| Portugal | 54 | 1592 | 1646 | 3.28 | 72 | 0.00249 |
| Singapore | 158 | 3692 | 3850 | 4.1 | 82 | 0.00249 |
| Slovenia | 66 | 3901 | 3967 | 1.66 | 77 | 0.00249 |
| Solomon Islands | 35 | 50 | 85 | 41.18 | 22 | 0.47264 |
| South Korea | 266 | 15983 | 16249 | 1.64 | 73 | 0.00249 |
| Spain | 162 | 8626 | 8788 | 1.84 | 83 | 0.00249 |
| Sweden | 360 | 10813 | 11173 | 3.22 | 79 | 0.00249 |
| Switzerland | 69 | 4628 | 4697 | 1.47 | 79 | 0.00249 |
| Thailand | 136 | 1714 | 1850 | 7.35 | 65 | 0.02488 |
| USA | 1352 | 194535 | 195887 | 0.69 | 87 | 0.00249 |
| United Kingdom | 6665 | 43545 | 50210 | 13.27 | 83 | 0.00249 |

Range of dates considered: 2022-10-29 - 2023-01-27

There were 15,938 CH.1 sequences that matched the search term: CA.7, CH.1, CH.1.1, CH.1.1.1, CH.1.1.2, CV.1

CH.1 and CH.1.1 lineages were increasingly sampled in 38/41 countries where it had been sampled ≥ 10 times. They have the consensus form BA.2.75 +[R346T,K444T,L452R,F486S]

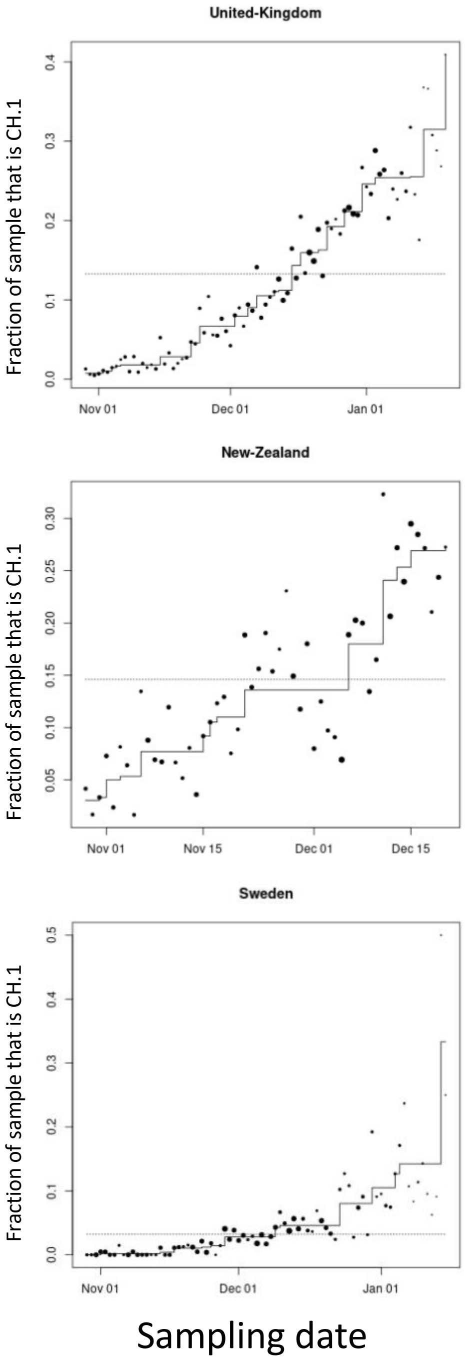


**Supplementary Figure 2. Isotonic regression analysis showing BA.2.75 sublineage CH.1 and related lineages are increasingly sampled over time in countries where they have become established.** The table provides summary statistics for all countries where CH.1 sequences have been sampled at least 10 times with a sampling date between Oct 29, 2022 and Jan 27, 2023. Three examples of the data over time are plotted to illustrate the increasing frequency of CH.1 sampling. The proportion of CH.1-related lineages in the total sample (y-axis) is calculated each day samples are available (x-axis). The size of the dot reflects the relative sample size on a given day. The *p*-value is calculated based on a one-sided resampling test with 400

randomizations. The results can be updated online using the Isotonic Regression tool at cov.lanl.gov. (https://cov.lanl.gov/content/sequence/ISORG/pango_isorg.html)[26]. CH.1 and BN.1 (Supplementary Fig. 3) related forms were chosen for display because they are currently the mostly commonly sampled BA.2.75 sublineages.

| Countries | Number of BN.1 sequences | Number of other sequences | Total number of seuqences | BN.1/Total (%) | Number of days sampled | One sided p-value BN.1 increasing |
|---|---|---|---|---|---|---|
| Australia | 1973 | 17737 | 19710 | 10.01 | 82 | 0.00249 |
| Austria | 720 | 30199 | 30919 | 2.33 | 82 | 0.00249 |
| Belgium | 125 | 3645 | 3770 | 3.32 | 81 | 0.1393 |
| Brazil | 78 | 9367 | 9445 | 0.83 | 70 | 0.00249 |
| Brunei | 43 | 727 | 770 | 5.58 | 61 | 0.33831 |
| Cambodia | 122 | 74 | 196 | 62.24 | 54 | 0.26617 |
| Canada | 452 | 32295 | 32747 | 1.38 | 83 | 0.00249 |
| Chile | 28 | 2898 | 2926 | 0.96 | 80 | 0.81841 |
| China | 40 | 6215 | 6255 | 0.64 | 83 | 0.86567 |
| Colombia | 41 | 1080 | 1121 | 3.66 | 57 | 0.45522 |
| Croatia | 34 | 1268 | 1302 | 2.61 | 55 | 0.00249 |
| Czech Republic | 78 | 1057 | 1135 | 6.87 | 71 | 0.00249 |
| Denmark | 4151 | 29245 | 33396 | 12.43 | 83 | 0.00249 |
| Finland | 36 | 2568 | 2604 | 1.38 | 73 | 0.35572 |
| France | 619 | 24387 | 25006 | 2.48 | 76 | 0.00249 |
| Georgia | 53 | 86 | 139 | 38.13 | 32 | 0.00249 |
| Germany | 2001 | 36712 | 38713 | 5.17 | 80 | 0.00249 |
| Greece | 99 | 2955 | 3054 | 3.24 | 56 | 0.00249 |
| Hong-Kong | 23 | 692 | 715 | 3.22 | 72 | 0.00249 |
| Iceland | 177 | 1004 | 1181 | 14.99 | 81 | 0.28856 |
| India | 88 | 1301 | 1389 | 6.34 | 76 | 0.6194 |
| Indonesia | 240 | 4952 | 5192 | 4.62 | 77 | 0.83831 |
| Iran | 24 | 84 | 108 | 22.22 | 32 | 0.45274 |
| Ireland | 315 | 4955 | 5270 | 5.98 | 85 | 0.00249 |
| Israel | 287 | 5367 | 5654 | 5.08 | 64 | 0.01741 |
| Italy | 237 | 9831 | 10068 | 2.35 | 83 | 0.00249 |
| Japan | 3582 | 62568 | 66150 | 5.41 | 87 | 0.00249 |
| Kenya | 10 | 226 | 236 | 4.24 | 39 | 0.53483 |
| Laos | 25 | 16 | 41 | 60.98 | 24 | 0.00249 |
| Latvia | 69 | 1126 | 1195 | 5.77 | 46 | 0.00249 |
| Liechtenstein | 10 | 140 | 150 | 6.67 | 55 | 0.75871 |
| Lithuania | 53 | 474 | 527 | 10.06 | 35 | 0.00249 |
| Luxembourg | 145 | 3592 | 3737 | 3.88 | 79 | 0.00249 |
| Malaysia | 454 | 3273 | 3727 | 12.18 | 79 | 0.76368 |
| Mauritius | 206 | 231 | 437 | 47.14 | 33 | 0.00249 |
| Mexico | 59 | 1781 | 1840 | 3.21 | 59 | 0.28358 |
| Moldova | 10 | 36 | 46 | 21.74 | 28 | 0.70647 |
| Netherlands | 514 | 8528 | 9042 | 5.68 | 80 | 0.0199 |
| New Zealand | 220 | 4751 | 4971 | 4.43 | 53 | 0.00249 |
| Norway | 33 | 1214 | 1247 | 2.65 | 70 | 0.39801 |
| Peru | 35 | 4751 | 4786 | 0.73 | 63 | 0.47761 |
| Poland | 35 | 792 | 827 | 4.23 | 74 | 0.00249 |
| Portugal | 51 | 1595 | 1646 | 3.1 | 72 | 0.00995 |
| Puerto-Rico | 31 | 899 | 930 | 3.33 | 49 | 0.64925 |
| Romania | 28 | 178 | 206 | 13.59 | 50 | 0.16915 |
| Russia | 90 | 4103 | 4193 | 2.15 | 50 | 0.00249 |
| Singapore | 358 | 3492 | 3850 | 9.3 | 82 | 0.00249 |
| Slovakia | 13 | 427 | 440 | 2.95 | 62 | 0.18657 |
| Slovenia | 107 | 3860 | 3967 | 2.7 | 77 | 0.00249 |
| South-Africa | 11 | 1096 | 1107 | 0.99 | 75 | 0.22139 |
| South-Korea | 3104 | 13145 | 16249 | 19.1 | 73 | 0.00249 |
| Spain | 159 | 8629 | 8788 | 1.81 | 83 | 0.96269 |
| Sweden | 401 | 10772 | 11173 | 3.59 | 79 | 0.00249 |
| Switzerland | 176 | 4521 | 4697 | 3.75 | 79 | 0.15423 |
| Thailand | 1225 | 625 | 1850 | 66.22 | 65 | 0.00249 |
| Trinidad and Toba | 75 | 270 | 345 | 21.74 | 75 | 0.00249 |
| USA | 5522 | 190365 | 195887 | 2.82 | 87 | 0.00249 |
| United Kingdom | 3250 | 46960 | 50210 | 6.47 | 83 | 0.00249 |
| Vietnam | 215 | 73 | 288 | 74.65 | 53 | 0.00498 |

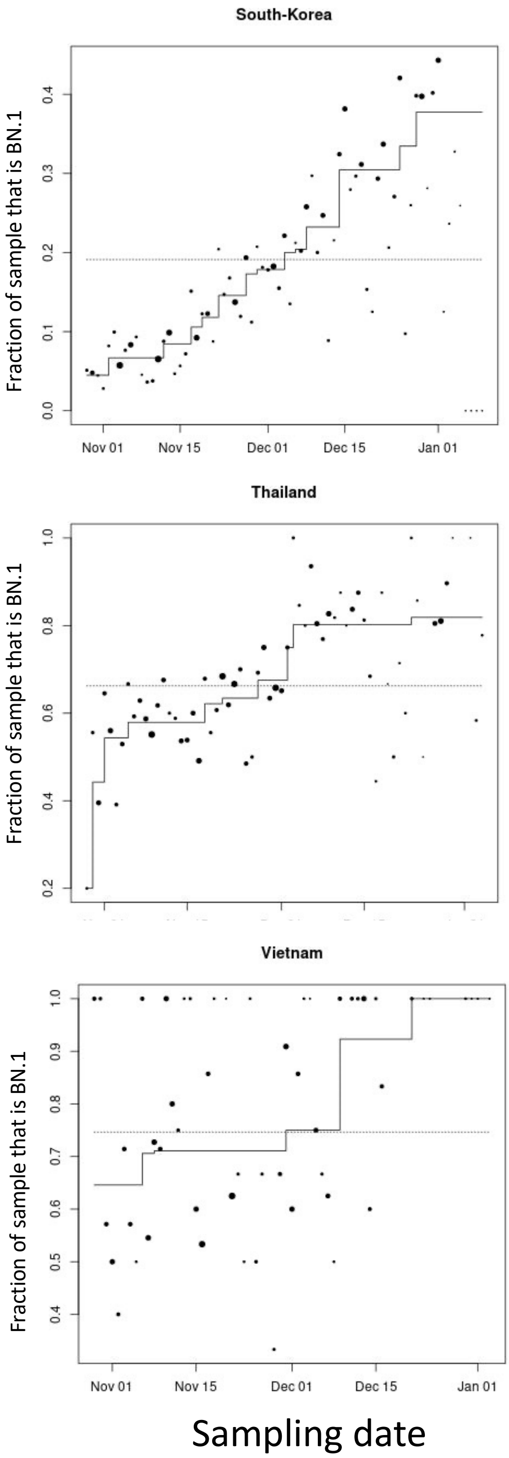


Range of dates considered: 2022-10-29 - 2023-01-27

There were 32,568 BN.1 sequences that matched the search term: BN.1, BN.1.1, BN.1.1.1, BN.1.2, BN.1.2.1, BN.1.3, BN.1.3.1, BN.1.4, BN.1.5, BN.1.6, BN.1.7, BN.1.8, BN.1.9

BN.1 and BN.1.1 lineages were increasingly sampled in 35/59 countries where they had been sampled ≥ 10 times. They have the consensus form BA.2.75 +[R346T,K356T,F490S]

**Supplementary Figure 3. Isotonic regression analysis showing BA.2.75 sublineage BN.1 and related forms are increasingly sampled over time in countries where they have become established.**

This figure follows the format of Supplementary Fig. 2, but here the table provides summary statistics for all countries where BN.1 sequences have been sampled at least 10 times between Oct 29, 2022 and Jan 27, 2023. As before, three examples are plotted to illustrate the increasing frequency of BN.1 sampling, and the proportion of BN.1-related lineages in the total sample (y-axis) is calculated each day samples are available (x-axis). The size of the dot reflects the relative sample size on a given day. The *p*-value is calculated based on a one-sided resampling test with 400 randomizations. The results can be updated online using the Isotonic Regression tool at cov.lanl.gov. (https://cov.lanl.gov/content/sequence/ISORG/pango_isorg.html)[26]. As noted above, CH.1 and BN.1 (Supplementary Fig. 2) were highlighted because they are currently the mostly commonly sampled BA.2.75 sublineages.

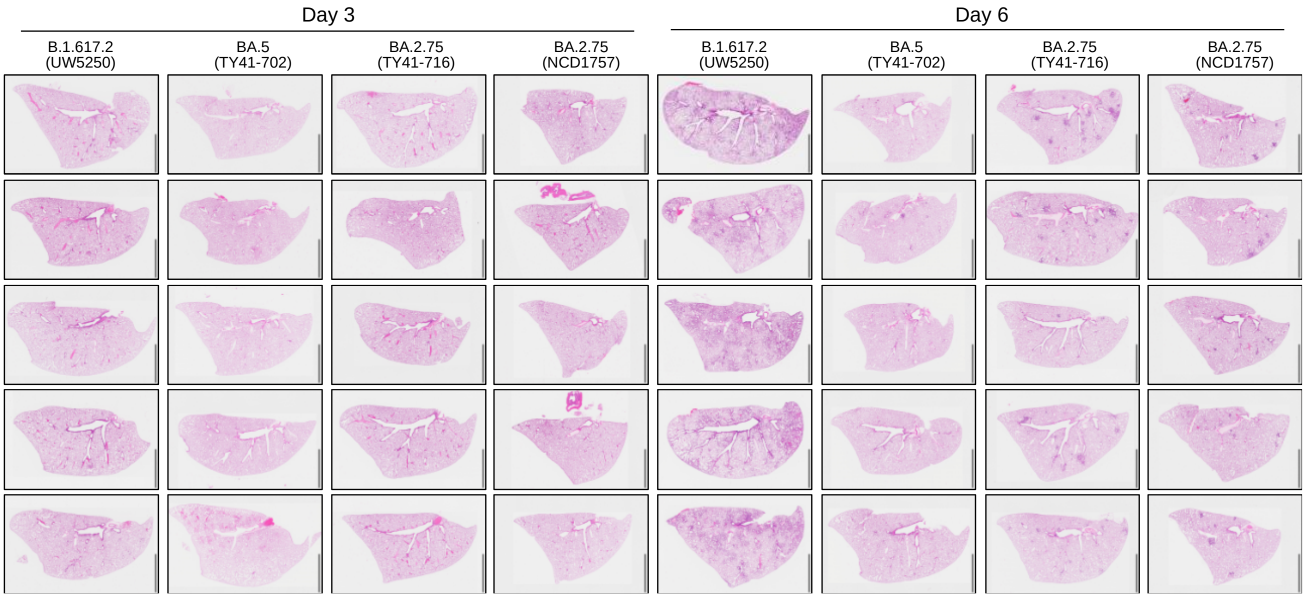


**Supplementary Figure 4. Semi-macroscopic images of the lungs of hamsters inoculated with SARS-CoV-2.**

Wild-type Syrian hamsters ($n$ = 5, per group) were inoculated with $10^5$ PFU of BA.2.75 (TY41-716), BA.2.75 (NCD1757), BA.5 (TY41-702), or B.1.617.2 (UW5250) and sacrificed at 3 or 6 dpi for histopathological examinations. Semi-macroscopic images (hematoxylin and eosin staining) of the lungs from all animals examined are shown. Scale bars, 5 mm. Data are from one experiment.

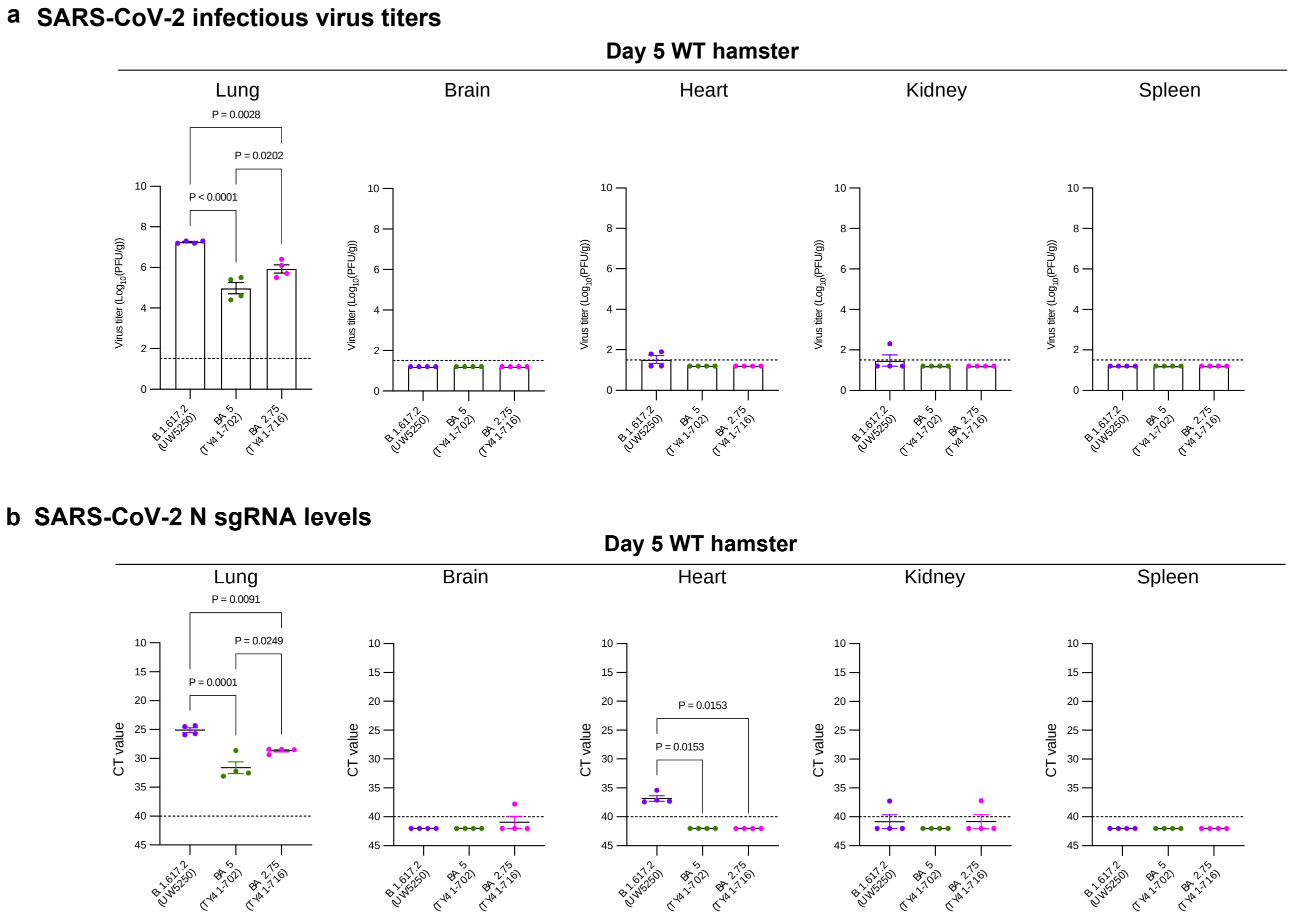


**Supplementary Figure 5. Viral replication in lungs and extrapulmonary organs of wild-type hamsters inoculated with BA.2.75.**

Wild-type Syrian hamsters ($n$ = 4) were inoculated with $10^5$ PFU of BA.5 (TY41-702), BA.2.75 (TY41-716), or B.1.617.2 (UW5250). Viral titers (**a**) and levels of subgenomic RNA (sgRNA) targeting the N gene (**b**) in the lungs, brain, heart, kidneys, and spleen of infected animals were measured at 5 dpi. Viral titers and sgRNA levels in the organs were determined by performing plaque assays and quantitative real-time PCR, respectively. Data are means ± s.e.m. Points represent data from individual hamsters; the lower limit of detection is indicated by the horizontal dashed line. Data were analyzed with the Kruskal–Wallis test with Dunn's multiple comparisons (titers and sgRNA levels in the extrapulmonary organs of infected hamsters) or a one-way ANOVA with Tukey's multiple comparisons test (titers and sgRNA levels in the lungs of infected hamsters).

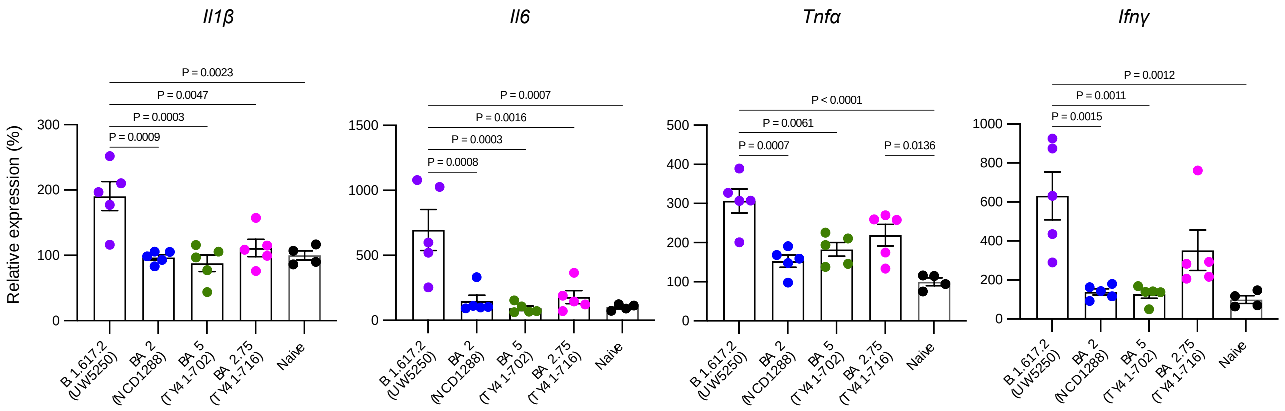


**Supplementary Figure 6. Host responses in the lungs of wild-type hamsters infected with BA.2.75.** Wild-type Syrian hamsters ($n$ = 5) were intranasally inoculated with $10^5$ PFU in 30 μL of BA.2 (NCD1288), BA.5 (TY41-702), BA.2.75 (TY41-716), or B.1.617.2 (UW5250). Pro-inflammatory cytokine gene expression levels (*Il1β*, *Il6*, *Tnfα*, and *Ifnγ*) in the lungs of the infected hamsters were assessed at 3 dpi (infected hamsters, $n = 5$; naïve uninfected hamsters, $n = 4$). Vertical bars show the mean ± s.e.m. Points indicate data from individual mice. Data were analyzed by using a one-way ANOVA with Tukey's multiple comparisons test. Data are from one experiment.
